# MicroRNA-143-3p and miR-452-5p: A Fingerprint for the Diagnosis of Aortic Stenosis in the Geriatric Population

**DOI:** 10.3390/biomedicines13030671

**Published:** 2025-03-10

**Authors:** Mónica Ramos, Francisco Javier Enguita, Fernando Bonet, Rocío Ayala, Francisco Javier Gómez-Pavón, Oscar Campuzano, Rocío Toro, Maribel Quezada-Feijoó

**Affiliations:** 1 Cardiology Department, Hospital Central de la Cruz Roja, 28003 Madrid, Spain; rayalamunoz@gmail.com (R.A.); maribelquezada2000@gmail.com (M.Q.-F.); 2 Medicine School, Alfonso X el Sabio University, 28007 Madrid, Spain; javiergomezpav@gmail.com; 3 Instituto de Medicina Molecular João Lobo Antunes, Faculty of Medicine, Lisbon University, Av. Prof. Egas Moniz, 1649-028 Lisbon, Portugal; fenguita@medicina.ulisboa.pt; 4 Research Unit, Biomedical Research and Innovation Institute of Cadiz (INiBICA), Puerta del Mar University Hospital, 11009 Cádiz, Spain; fbonetmartinez@gmail.com; 5 Geriatrics Department, Hospital Central de la Cruz Roja, 28003 Madrid, Spain; 6 Medical Science Department, School of Medicine, University of Girona, 17003 Girona, Spain; oscar@brugada.org; 7 Institut d’Investigació Biomèdica de Girona (IDIBGI-CERCA), 17190 Salt, Spain; 8 Centro Investigación Biomèdica en Red, Enfermedades Cardiovasculares (CIBERCV), 28029 Madrid, Spain; 9 Medicine Department, School of Medicine, University of Cadiz, 11003 Cádiz, Spain

**Keywords:** aortic stenosis, RNA sequencing, microRNA, geriatric population

## Abstract

**Background/Objectives:** Aortic stenosis (AS) is the most common valvular pathology in the geriatric population and is the primary cause of valve replacement. However, misdiagnoses and delays in treatment are common due to comorbidities, frailty, and sedentary lifestyles among elderly individuals. MicroRNAs (miRNAs) are highly conserved molecular regulators involved in various cellular processes and have gained recognition as reliable biomarkers in cardiovascular diseases. In the present study, we evaluated plasma miRNAs as potential biomarkers for the early diagnosis of AS in the geriatric population to identify early therapeutic strategies. **Methods:** This prospective, case–control study included 87 individuals over 75 years of age. The participants were divided into AS (n = 58) and control (n = 29) groups. **Results:** Fifty-four miRNAs were differentially expressed between patients with AS and controls. Among those genes, 29 were upregulated and 25 were downregulated in patients with AS relative to controls. We selected seven candidate genes (miR-185-5p, miR-143-3p, miR-370-3p, let-7d-3p, miR-452-5p, miR-6787-3p, and miR-21-3p) for experimental validation by qRT–PCR. Only miR-143-3p and miR-452-5p were significantly upregulated in the plasma of patients with AS compared with controls. We developed a multiparametric model by combining the two-miRNA signature with echocardiographic parameters (left ventricular ejection fraction, stroke volume, and global longitudinal strain) to increase diagnostic power; this model yielded sensitivity, specificity, and area under the receiver operating characteristic curve (AUC) values of 78.2%, 70.7%, and 0.837, respectively. **Conclusions:** In clinical practice, the use of a multiparametric model involving this set of miRNAs combined with echocardiographic variables may improve the accuracy of AS diagnosis and risk stratification.

## 1. Introduction

Aortic stenosis (AS) is the most frequently diagnosed primary valvular disease and represents the leading indication for valve replacement [1]. The pathophysiology of AS development involves a combination of inflammatory processes, endothelial dysfunction, and osteogenic differentiation of valvular interstitial cells (the process by which quiescent resident valve interstitial cells undergo activation and redifferentiate to an osteoblast-like phenotype), which ultimately lead to progressive calcification and valvular obstruction [2]. AS is one of the most important causes of congestive heart failure (HF), morbidity, and hospitalization in elderly patients; moreover, its late diagnosis is associated with its poor prognosis [3,4]. There is no drug-based treatment to prevent osteogenic processes. Aortic valve replacement (AVR) or transcatheter aortic valve implantation are invasive and definitive therapeutic options. As a rule, the identification of symptomatic patients with AS is based on warning signs such as chest pain, syncope, and dyspnoea [5,6]. However, misdiagnoses and delays in treatment are common due to comorbidities, frailty, and sedentary lifestyles among elderly individuals [7,8]. This situation results in irreversible myocardial damage associated with diastolic dysfunction, left ventricular impairment, pulmonary hypertension, and premature death.

Cardiologists diagnose AS based on imaging tests and traditional biomarkers used for the general population. In accordance with the guidelines set forth by the European Society of Cardiology (ESC), the use of the B-type natriuretic peptide (BNP) and N-terminal pro-brain natriuretic peptide (NT-proBNP) is recommended [9]. However, these biomarkers lack sufficient precision; they do not reflect the consequences of severe AS, and their plasma levels vary with age, atrial fibrillation (AF) onset, or renal failure [10,11]. Specific parameters for identifying elderly patients at risk of developing cardiac fibrosis and ventricular dysfunction, as well as rapid calcification, are currently unavailable. However, novel biomarker research may prove beneficial for this population by facilitating optimized diagnoses and risk assessments in the initial phase for asymptomatic patients with AS [12,13,14].

MicroRNAs (miRNAs) are small noncoding RNAs (ncRNAs) that act by inhibiting the translation of messenger RNAs (mRNAs) into proteins [15]. These miRNAs play critical roles in most cellular functions, including metabolism, proliferation, and cell signaling [16]. miRNAs regulate gene expression and may play essential roles in inflammation and osteogenic differentiation in the AS process [17,18]. Numerous studies have indicated that miRNA dysregulation may result in impaired gene expression in the context of AS [19,20,21]. miR-29b has been shown to activate the Wnt3/β-catenin/Smad3 signaling pathway, leading to the inhibition of TGF-β3 and, consequently, the promotion of aortic valve calcification [19]. miR-638 targets Sp7, thereby inhibiting this process. miR-664a-3p, which is downregulated in AS, influences the osteogenic differentiation and calcification of the aortic valve through BMP2 [20]. Chen et al. [21] demonstrated that osteogenesis was facilitated through miR-101-3p, which targeted the cadherin-11 and Sry-related high-mobility group box 9 (SOX9) pathways, both of which are pivotal for osteogenesis. Despite recent advances in our understanding of the pathological process of AS, there is still a need for improved diagnostic tools that can accurately identify this condition across various populations. Factors such as frailty, nonmobility, comorbidities, the lack of specific biomarkers, and drug-based therapies present challenges in developing a severe AS diagnostic tool that aligns with current ESC guidelines. It is expected that in the coming years, due to the aging of the population, the number of AS cases will increase. Although echocardiography is an accessible test, some geriatric patients have mobility limitations, so the use of biomarkers that raise suspicion of AS could save complicated and unnecessary movements. However, there are limitations to echocardiographic diagnosis, for example, in the case of poor acoustic echocardiographic windows or in those stenoses that are at the limit of severity, when they have low gradients and there are doubts as to whether they are moderate or severe, requiring complementary tests such as a CT scan for calcium score quantification. For this reason, the use of other biomarkers that can support the diagnosis and even serve as future therapeutic targets is highly important. The objective of this study was to develop a plasma miRNA signature that can identify severe AS among elderly patients, thereby improving the accuracy of traditional echocardiographic diagnosis.

## 2. Materials and Methods

### 2.1. Study Sample

This was a prospective, observational, case–control study. Patients were recruited consecutively between February 2022 and January 2023 from the outpatient clinic of the Hospital Cruz Roja, Madrid (Spain). A total of 87 individuals over 75 years of age were included and divided into two cohorts: (i) those diagnosed with severe degenerative AS (n = 58) and (ii) healthy age- and sex-matched controls (n = 29). Patients with moderate AS or moderate–severe concomitant valve impairments or individuals who were under 75 years old were excluded. As controls, 29 subjects were identified from a list of outpatients attending our hospital for the treatment of hypertension, dyslipidaemia, or arrhythmia and who showed no evidence of AS in their history or physical examinations or moderate–severe concomitant valve impairment, cardiomyopathies, or coronary artery disease. The subjects were stratified by sex and age to match each patient with AS.

Severe AS was indicated by a peak velocity (ΔVPeak) greater than 4 m/s, a mean pressure gradient (MPG) greater than 40 mmHg, and a dimensionless index (DI) less than 0.25. Severe AS with a low gradient was indicated by an aortic valve area (AVA) less than 1 cm and an indexed AVA lower than 0.6 cm/m^2^ [6,9].

Clinical and demographic baseline characteristics were collected. The Charlson comorbidity index was calculated [22]. Coronary artery disease (CAD) was defined as a documented history of acute coronary syndrome or coronary artery disease confirmed by coronary angiography or previous coronary revascularization [23].

The research protocol received approval from the Ethics Committee of Alfonso X el Sabio University (UAX) and adhered to the ethical principles outlined in the Declaration of Helsinki. Written informed consent was obtained from all participants.

### 2.2. Echocardiography

All patients underwent comprehensive transthoracic echocardiography on a commercially available Phillips Affinity-70C (Philips Ultrasound, Bothell, WA, USA) echocardiograph with an S5-1 probe. The echocardiogram was performed by an experienced echocardiographer (M.Q-F., M.R). The severity of the aortic valve disease was determined in accordance with the recommendations of the ESC by evaluating the ΔVPeak, the MPG (calculated using the simplified Bernoulli equation), the AVA, determined through the continuity equation, and the dimensionless index (DI), which reflects the ratio between the time–velocity integral of the left ventricular outflow tract (LVOT) and that of the aortic valve jet [6,9]. The left ventricle stroke volume (LV-SV) was calculated by multiplying the LVOT area by the LVOT time–velocity integral.

Global longitudinal strain (GLS) was assessed using 2D speckle tracking from apical 2-, 3-, and 4-chamber views, following the 17-segment left ventricular (LV) model established by the American Society of Echocardiography [24]. GLS was derived as the mean value of regional strain measurements. Analysis was performed using an automated function imaging software package.

### 2.3. Blood Collection

Peripheral blood samples (10 mL) were drawn into K2-EDTA tubes following a 10 h overnight fast. Samples were immediately centrifuged at 1500× *g* for 15 min at 4 °C, and plasma was separated within 4 h of collection. The supernatant was then aliquoted and stored at −80 °C until further analysis

### 2.4. RNA Isolation

Total RNA was isolated from 200 µL of plasma using the miRNeasy Serum/Plasma Advanced Kit (Qiagen, Hilden, Germany) in accordance with the manufacturer’s guidelines. During extraction, 3.5 µL of miRNeasy Serum/Plasma Spike-In Control (1.6 × 10^8^ copies/µL of the C. elegans miR-39 miRNA mimic) was added to each sample as an internal control. RNA was eluted in 20 µL of RNase-free water and quantified using a Qubit RNA High-Sensitivity Assay Kit with a Qubit^®^ 2.0 fluorometer (Life Technologies, Carlsbad, CA, USA). For miRNA expression analysis, 5 ng of RNA was reverse transcribed using the miRCURY LNA RT Kit (Qiagen, Hilden, Germany), following the manufacturer’s protocol.

### 2.5. RNA Sequencing Analysis and Bioinformatics

Standard miRNA libraries were generated using a NEBNext Multiplex Small RNA Library Prep Set for Illumina (Set 1) (New England Biolabs, Ipswich, MA, USA) following the manufacturer’s recommendations, and single-end libraries were sequenced on an Illumina SE75 Platform with an output of approximately 20 M reads per sample.

For miRNAs, raw data (raw reads) in fastq format were filtered and trimmed for primer sequencing with Trimmomatic 0.39 software [25]. miRNA sequences were detected using the STAR aligner included within the miRMaster 2.0 software suite [26]. Analysis of differentially expressed miRNAs was performed using RNfuzzyApp v1 software [27]. The miRNAs that were differentially expressed were defined as those meeting the criteria 1.0 > Log2-fold change (FC) > 1.0. The use of a cutoff based on a log2-fold change (LFC) > 1.0 or <−1.0 to determine differential expression of microRNAs (miRNAs) is a widely accepted standard in transcriptomics analysis. This threshold helps identify biologically significant changes while minimizing technical and statistical noise. In the context of gene regulation, a change of this magnitude typically reflects a biologically relevant effect on cellular processes. These values have been broadly adopted in transcriptomics studies [28].

### 2.6. Validation of miRNA Profiles

For the validation study, the selected miRNA candidates were quantified using miRCURY LNA miRNA Custom PCR Panels (Qiagen, Hilden, Germany). Reverse transcription was carried out with the miRCURY LNA RT Kit (Qiagen, Hilden, Germany), followed by qRT-PCR using the miRCURY LNA SYBR Green PCR Kit (Qiagen, Hilden, Germany), as previously described [29]. Amplification curves were analyzed with CFX Manager™ 3.1 software (Bio-Rad, Hercules, CA, USA). To ensure accurate normalization, the most stably expressed reference miRNAs, miR-28-5p and miR-126-3p, were identified using the NormFinder algorithm [29]. Normalization was performed using the ΔCq method, where ΔCq = mean (CqmiR-28-5p and miR-126-3p)—Cq miRNA. Prior to statistical analysis, miRNA levels were log-transformed. The specificity of amplification was verified through melting curve analysis.

### 2.7. GO Term Enrichment Analysis

The miRWalk (http://mirwalk.umm.uni-heidelberg.de/, accessed from the 20 September 2024 database) was utilized to predict target genes, construct the miRNA-mRNA regulatory network, and conduct gene ontology (GO) term analysis. GO analysis was performed to assess the involvement of the two differentially expressed miRNAs in biological processes, cellular components, and molecular functions, predict the targeted genes, construct the miRNA–mRNA regulatory network, and conduct gene ontology (GO) term analysis. 

### 2.8. Statistical Analysis

Continuous variables are presented as means ± standard deviations. Categorical variables are shown as frequencies and percentages (%). Outliers were detected using the Rout method, with Q = 1% [30]. The normality of each variable was assessed using the Shapiro–Wilk test. Comparisons of miRNA levels between groups were conducted using a Student’s *t*-test (for normally distributed variables) or nonparametric Mann–Whitney and Kruskal–Wallis tests (for non-normally distributed variables). Differences between groups were analyzed using analysis of variance. Receiver operating characteristic (ROC) curves were created to evaluate the diagnostic performance of the candidate miRNAs, and logistic regression models were built to calculate the area under the curve (AUC) and determine the specificity and sensitivity of the optimal cutoff values. The optimal cutoff points for each miRNA were determined using the Youden Index, which maximizes the sum of the sensitivity and specificity. These cutoff values were then applied to calculate diagnostic accuracy, sensitivity, and specificity for each miRNA individually and in combination. ROC curves were created by plotting sensitivity against 1−specificity. The results are presented as the AUC along with the 95% confidence interval. The variations in the *p*-values of the variables were assessed using the Wald test and likelihood ratio. All analyses were performed using the R statistical software package (Team RC. R: A Language and Environment for Statistical Computing. https://www.r-project.org, accessed on 15 March 2024).

## 3. Results

The baseline characteristics of the study population are shown in Table 1. We enrolled 87 participants, including 58 patients with severe AS and 29 healthy individuals. There were no statistically significant differences in sex or age between the two groups. The presence of AF was statistically significant in patients with AS. The echocardiographic data are presented in Table 1. Statistically significant differences were found in the ejection fraction (EF) estimated by the Simpson method or GLS between cohorts. When considering the analytical profile and medications, only the use of diuretics and the NT-proBNP levels were significantly different (*p* < 0.001).

### 3.1. Circulating miRNA Expression Profiles

**Figure 1:**
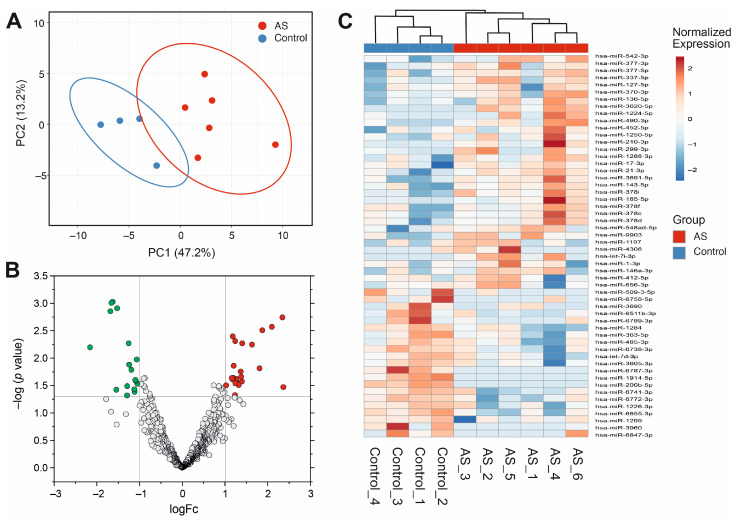
Exploratory analysis of differentially expressed miRNAs between patients with severe aortic stenosis and controls as determined by miRNA sequencing. (**A**) Volcano plot of miRNAs, highlighting those miRNAs not statistically significant (FDR > 0.05 and absolute FC < 2 (abslog2FC < 1) in bold blue); miRNAs downregulated (abslog2FC > −1) in green; and miRNAs upregulated (abslog2FC > 1) in red. (**B**) The 3D principal component analysis plot of miRNA expression in patients with severe aortic stenosis (n = 6, in red, left) and controls (n = 4, in blue). (**C**) Expression heatmap with hierarchical clustering of controls on the left side in blue and the severe aortic stenosis cohort on the right side in red. Abbreviations: miRNAs, microRNAs; FC, fold change; FDR, false discovery rate.

RNA-seq screening was performed in a cohort of six AS patients and four controls to identify potential plasma miRNAs associated with AS. Principal component analysis (PCA) was employed to visualize the clustering of samples based on the genes with the highest variability in expression, and no outliers were detected. PCA revealed a clear distinction between the control and AS samples in terms of miRNA profiling (Figure 1B). The RNA-seq produced an average of 8.6 million reads per sample. The filtered RNA-seq read set identified 698 plasma circulating miRNAs in total (Table S1). On average, miR-26a-5p was the most abundant miRNA in both AS patients and healthy controls. The top ten most abundant miRNAs detected in these two groups are listed in Table S2. In total, 54 miRNAs were differentially expressed between AS patients and controls, with a −1.0 > Log2-fold change (FC) > 1.0. Among those genes, 29 were upregulated and 25 were downregulated in AS patients relative to controls (Figure 1A,C).

### 3.2. Differentially Expressed Circulating miRNAs in the AS Cohort

**Figure 2:**
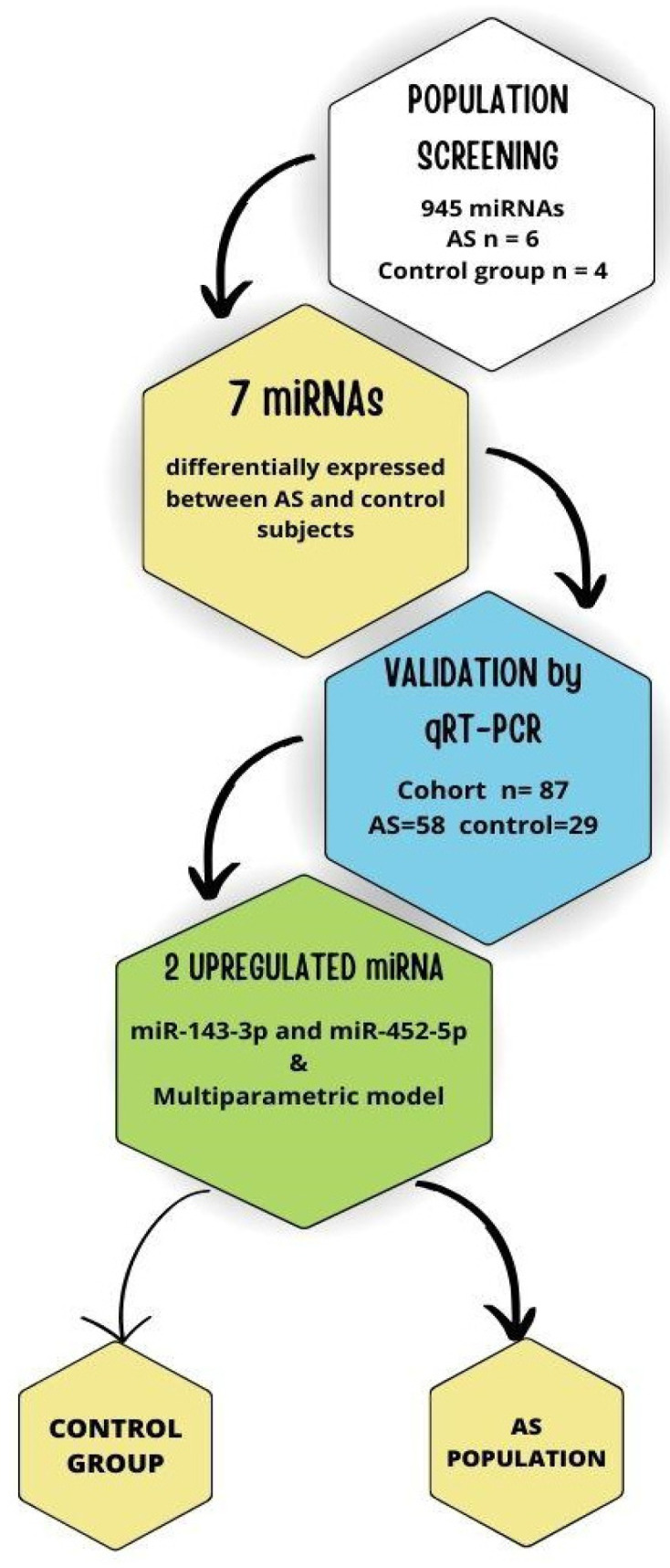
Flowchart of the study design. This figure illustrates the experimental workflow of the study, including screening and validation. Abbreviations: AS, aortic stenosis; miRNA, microRNA.

**Figure 3:**
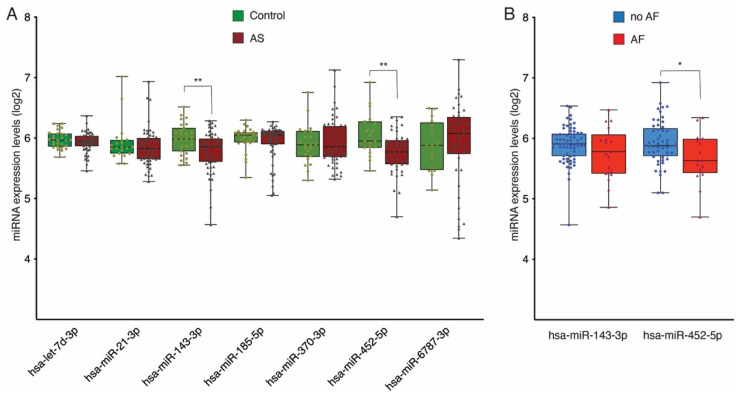
Boxplots of miRNA expression levels. (**A**) miRNA expression levels in the AS and control cohorts. (**B**) miRNA expression levels the no AF and AF groups. The analysis was carried out by qPCR. The data are presented as log2 values. The data represent the means ± SEMs. * *p* < 0.05, ** *p* < 0.01. The error bars represent SDs. AS, aortic stenosis; AF, atrial fibrillation.

The flowchart of the study design is shown in Figure 2. Among the 54 differentially expressed miRNAs, we selected 7 candidates (miR-185-5p, miR-143-3p, miR-370-3p, let-7d-3p, miR-452-5p, miR-6787-3p, and miR-21-3p) for experimental validation by qRT–PCR in all the recruited patients with AS (n = 58) and healthy controls (n = 29). The selection of these candidates was based on two main criteria: (1) the miRNAs with the highest reads per million (RPM) values in the RNA sequencing analysis, highlighting their relative abundance in plasma samples, and/or (2) those exhibiting the largest differences in expression levels (log-fold change) between aortic stenosis (AS) patients and healthy controls. These criteria ensured that the selected miRNAs were both statistically significant and biologically relevant for further investigation. For data normalization, miR-126-3p and miR-28-5p were used as housekeeping miRNAs, as they were the most stably expressed pair of miRNAs according to the NormFinder algorithm (15289330). Compared with those in the controls, only miR-143-3p and miR-452-5p were significantly upregulated in the plasma of AS patients (Figure 3A).

We next investigated the correlation between the differentially expressed miRNAs and several significant clinical parameters in the AS group. No correlations were detected between miR-143-3p or miR-452-5p expression and echocardiographic parameters such as the indexed AVA, MPG, peak aortic pressure gradient (PPG), ΔVPeak, or GLS. Additionally, to assess whether AF influences miRNA expression, we compared the expression levels of miR-143-3p and miR-452-5p between patients with and without AF. A Mann–Whitney U test showed no significant differences for miR-143-3p. However, a Student’s *t*-test revealed a slight but significant difference in miR-452-5p expression between groups (*p* = 0.02) (Figure 3B). However, the absolute difference in expression levels was small (log2 FC = 5.9 vs. 5.6).

### 3.3. miR-143-3p and miR-452-5p as Biological Markers of AS

**Figure 4:**
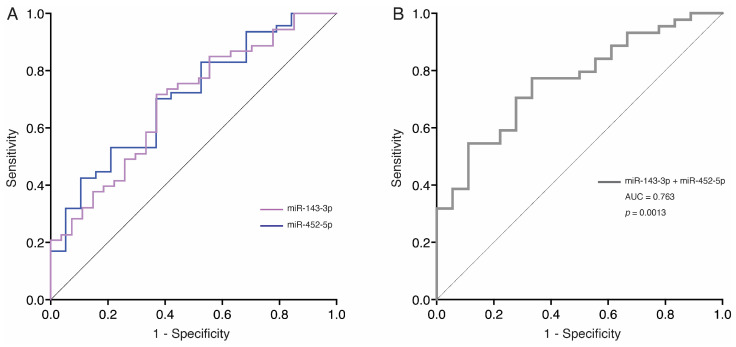
ROC curves for evaluating the predictive performance of differentially expressed miRNAs to discriminate between AS patients and controls. (**A**) ROC curves for miR-143-3p and miR-452-5p; (**B**) ROC curve of the two-miRNA panel combination value of miR-143-3p and miR-452-5p. AUC: area under the curve; AS, aortic stenosis.

We assessed the ability of the differentially expressed miRNAs to discriminate between patients with AS and healthy controls via the AUC. As shown in Figure 4A, both miR-143-3p and miR-452-5p displayed moderate performance, with AUC values of 0.690 (95% CI: 0.569–0.812; *p* = 0.006) and 0.707 (95% CI: 0.572–0.842; *p* = 0.009), respectively. The optimal cutoff points for each miRNA were determined to assess their diagnostic accuracy: For miR-143-3p, the cutoff of >0.3965 yielded a sensitivity of 85.2% and a specificity of 98.1%. For miR-452-5p, the cutoff of >0.4165 showed a sensitivity of 85.2% and a specificity of 96.2%. Then, we considered the diagnostic potential of the two-miRNA signature to distinguish between patients with AS and healthy individuals. The AUC for the combination of miR-143-3p and miR-452-5p was 0.763 (95% CI: 0.640–0887; *p* = 0.001), indicating improved discriminatory power (Figure 4B). The sensitivity, specificity, and accuracy of each miRNA and the two-miRNA signature are shown in Table 2.

**Figure 5:**
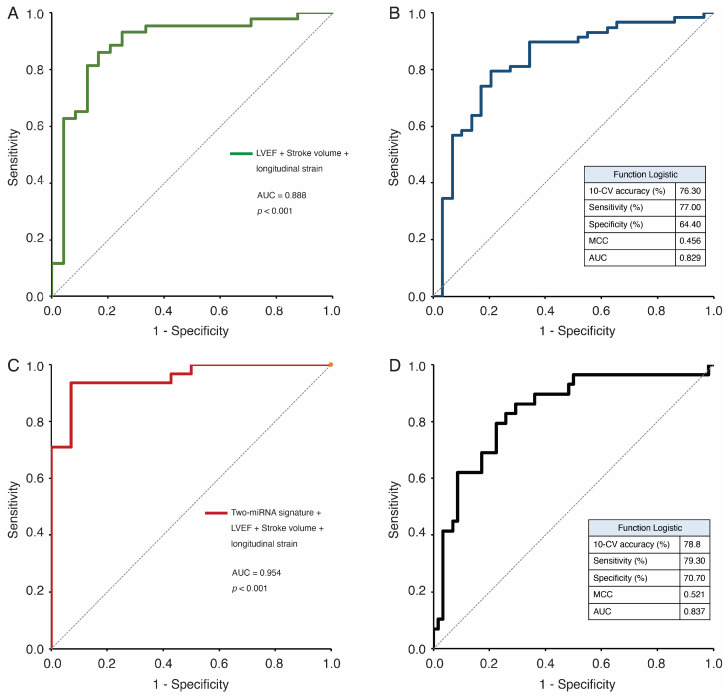
ROC curves for evaluating the predictive performance of clinical factors with differentially expressed miRNAs. (**A**) ROC curve of the clinical-only model (GLS + LVEF + LV-SV). (**B**) ROC curve of the 10-fold cross-validation test for the clinical-only model. (**C**) ROC curve of the combined value of the 2-miRNA panel +LVEF + LV-SV + GLS model. (**D**) ROC curve of the 10-fold cross-validation test for the 2-miRNA panel + GLS + LVEF + LV-SV model (function logistic). GLS, global longitudinal strain; LVEF, left ventricular ejection fraction; LV-SV, left ventricular stroke volume; MCC, Matthews correlation coefficient.

To further assess the diagnostic potential, we first evaluated a clinical-only model based on echocardiographic parameters (GLS, LVEF, and LV-SV). This model achieved an AUC of 0.887, with a sensitivity of 84.21%, specificity of 83.33%, and an overall accuracy of 83.58% (Figure 5A) (Table 3). Cross-validation using Weka confirmed these findings, with the function logistic algorithm achieving an accuracy of 76.30%, sensitivity of 77%, specificity of 64.4%, MCC of 0.456, and an AUC of 0.829 (Figure 5B).

We next developed a multiparametric model combining the differentially expressed miRNAs alongside echocardiographic parameters to increase the diagnostic power to discriminate patients with AS. Multivariate logistic regression analyses revealed that the combination of the two-miRNA signature plus LVEF, LV-SV, and GLS significantly improved the diagnostic ability of the two-miRNA signature, with an AUC value of 0.954 and an accuracy of 88.89% (Figure 5C). Next, we used the 10-fold cross-validation method via Weka to assess the predictive value of our model. After all the classifiers were run, the results indicated that the most successful algorithms were function logistic, which achieved a mean training accuracy, sensitivity, specificity, Matthews correlation coefficient (MCC), and an AUC of 78.8%, 79.30%, 70.70%, 0.521, and 0.837, respectively (Figure 5B). The sensitivity, specificity, and accuracy of each clinical parameter and the multiparametric model are shown in Table 3.

### 3.4. Gene Ontology Enrichment Analysis

**Figure 6:**
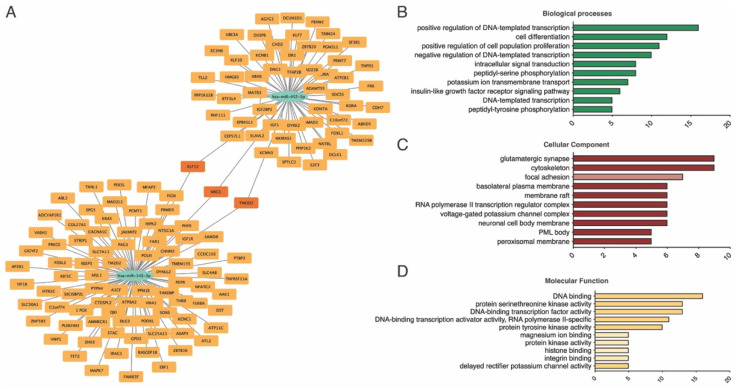
GO analysis of differentially expressed miRNAs. (**A**) miRNA–gene network for miR-143-3p and miR-452-5p; (**B**) GO BP functional enrichment analysis of miR-143-3p and miR-452-5p; (**C**) GO CC functional enrichment analysis of miR-143-3p and miR-452-5p; (**D**) GO MF functional enrichment analysis of miR-143-3p and miR-452-5p. GO, gene ontology; miRNA, microRNA; BP, biological process; CC, cellular component; MF, metabolic function.

We investigated the biological significance of the two differentially expressed miRNAs via GO enrichment analysis. We used the miRWalk database (http://mirwalk.umm.uni-heidelberg.de, accessed on 15 March 2024) to predict the putative targets of miR-143-3p and miR-452-5p. According to the miRWalk database, miR-143-3p was associated with 82 mRNAs, and miR-452-5p was associated with 61 mRNAs. Interestingly, three mRNA targets, KLF12, NRG1, and TMOD2, were shared by these two miRNAs (Figure 6).

We performed GO enrichment analysis of the differentially expressed miRNAs. The top 10 highly enriched GO terms of biological process (BP), cellular component (CC), and molecular function (MF) are shown in Figure 6B–D and Table 4, Table 5 and Table 6. The most enriched terms for BP, CC, and MF GO analysis were peptidyl-tyrosine phosphorylation, the neuronal cell body membrane and protein tyrosine kinase activity, respectively.

## 4. Discussion

We investigated the circulating miRNA signature of severe AS in a sample of 10 out of 87 elderly individuals. We identified two miRNAs that were upregulated in this cohort, namely, miR-143-3p and miR-452-5p. Moreover, when we added echocardiographic information to the model, the predictive accuracy of the model markedly increased.

In the field of AS research, miRNAs have also been implicated in the progression and establishment of aortic valve stenosis [31,32]. Coffey et al. [33] proposed a research project that included moderate to severe AS with and without coronary artery disease. They reported that miR-21-5p was overexpressed and that miR-22-3p was decreased in the plasma of a moderate to severe AS cohort without coronary artery disease. These authors subsequently demonstrated that miR-122-5p, miR-625-5p, and miR-30e-5p were downregulated and that miR-21-5p and miR-221-3p were upregulated in severe AS valvular tissue. MiR-21-5p has also been shown to be associated with left ventricular hypertrophy (LVH) and myocardial fibrosis [34]. In patients with severe AS, miR-19 expression was found to be decreased in both the plasma and myocardium, which is related to myocardial fibrosis. This miRNA has been linked to the extent of collagen fibril cross-linking, the lysis of oxidative proteins and the enzymes responsible for these processes [35]. Fabiani et al. [36] demonstrated the relationship of miRNA-21 with the presence of myocardial fibrosis in patients with AS. Derda et al. [37] proposed a peripherical blood study to discriminate among three different causes of LVH: hypertrophic cardiomyopathy, AS, and nonobstructive hypertrophic cardiomyopathy. miR-29c, which is involved in myocardial fibrosis, was upregulated in the AS cohort; interestingly, miR-29-c/miR29-a were proposed to discriminate between hypertrophic cardiomyopathy and AS [37]. Gabriel et al. [38] reported that tissue miR-4709-3p and cTNT-hs were independent predictors of hypertrophy regression, suggesting a potential opportunity for therapy. Takahashi et al. [39] demonstrated that miR-30c was overexpressed, whereas the levels of miR-106a, miR-148a, miR-204, miR-211, miR-31, and miR-424 were lower in the AS group than in the control group. MiR-30c is related to the number of osteogenic progenitor cells and osteocalcin protein [39]. Similarly, miR-214 was empirically confirmed to activate an inflammatory cascade of mediators that promote osteogenic differentiation [40]. According to Takahashi et al. [39], other authors have confirmed that, in AS valvular tissue, decreased levels of miR-204 are associated with osteoblastic biological progression. The authors proposed the synergistic effects of miR-204 and miR-486, which are overexpressed, on osteogenic differentiation [41]. Moreover, miR-204 has been proposed as a therapeutic target to inhibit calcification in degenerative AS [42]. Circulating miR-210 levels were greater in a cohort of elderly patients with AS and were also proposed as a risk stratification marker, thus providing additional information above and beyond NT-proBNP or echocardiographic parameters [43].

In our study, two miRNAs were enriched in elderly patients with AS compared with elderly controls. The role of miR-143 in smooth muscle cell biology has been previously established [44,45]. These findings may have direct implications for the biology of individual cells within aortic valves. Similarly, Fiedler et al. [31] evaluated the expression of multiple miRNAs in the aortic valves of patients with AS who were undergoing AVR. A comparison was made between the expression of these miRNAs in the aortic valves of healthy controls from a biobank. The results demonstrated elevated levels of miR-21, miR-24, and miR-143 in patients with AS. Subsequent experiments demonstrated that miR-143 regulates the expression of the matrix gla protein (MGP) via direct binding to its 3′UTR. Notably, MGP is involved in osteogenesis and plays a crucial role in maintaining homeostasis in aortic valves [46]. The potential of miR-143 to diagnose patients at an elevated risk of rapid calcification is a promising avenue for further investigation [31]. Such an approach could facilitate more rigorous monitoring and enable the implementation of optimal treatment strategies before the onset of ventricular complications associated with AS. Moreover, patients with a profile indicative of the rapid calcification of a mechanical prosthesis may prove optimal. Furthermore, future modulation of miR-143 may represent a potential therapeutic strategy for the prevention of aortic valve calcification.

MiR-452-5p is involved in various intracellular processes, including tumorigenesis and chronic contractile injury [47,48]. Li et al. [49] demonstrated significant upregulation of miR-452-5p in H9c2 cardiomyocytes after hypoxia exposure. The overexpression of miR-452-5p effectively mitigated the effects of circANKIB1 on hypoxia-induced H9c2 cell injury and ferroptosis, indicating that circANKIB1 protected cardiomyocytes from hypoxia by sponging miR-452-5p. In human cardiac fibroblasts, miR-452-5p was confirmed to target SMAD4, thereby influencing myocardial fibrosis [50].

Although AF was significantly more prevalent in patients with severe AS, probably related to increased LV filling pressures, we did not find a significant correlation between AF and the expression levels of miR-143-3p. In the study by Kiyosawa et al. [51], miRNA 143-3p was one of the miRNA which tended to show higher plasma levels in patients with high CHA2DS2-VASc scores and lower plasma levels in patients who underwent catheter ablation. The results suggested that the possible target genes were functionally associated with TGF-β signaling, which is a key pro-fibrotic element in various tissue and would contribute to atrial fibrosis. Given the established role of AF in cardiovascular remodeling, further studies with larger cohorts are warranted to determine whether AF may influence the expression of these specific miRNAs and to explore potential mechanistic links. On the other hand, slight but significant difference was observed in miR-452-5p expression (*p* = 0.02), but this difference was of small magnitude (log2 FC = 5.9 vs. 5.6) and does not necessarily indicate a strong association between AF and miR-452-5p levels. Importantly, miR-452-5p was also significantly upregulated in AS patients compared to controls, suggesting that its expression is more likely driven by AS-related remodeling processes rather than being exclusively associated with AF. Previous studies have indicated that AS and AF share common pathophysiological mechanisms, including fibrosis and inflammation, which may partially explain this finding [33]. However, further investigations are required to determine whether miR-452-5p plays a direct role in AF pathophysiology or if its differential expression in AF patients is a secondary effect of the underlying AS process.

In the review carried out by Mir et al. [52], they addressed the current situation regarding the regulation and pathophysiological functions of miRNAs in cardiovascular diseases (CVD) and possible future perspectives on their use as biomarkers for monitoring as well as therapeutic agents. A single biomarker appears to be insufficient to diagnose CAD or acute myocardial infarction; however, a combination of different miRNAs may be beneficial for the diagnosis and stratification of CVD. In the case of AF, selective therapies targeting miRNAs, such as upregulation and/or downregulation, have been studied to treat patients with AF. The results after pulmonary vein ablation can be improved through miRNA therapy, preventing cardiac electrical and fibrotic remodeling after AF ablation [53]. Studies have even been conducted in the field of HF [54,55]. Sardu et al. [54] proposed that in patients with a response to cardiac resynchronization therapy (CRT), there is a modulation of several miRNAs involved in the modulation of the processes of cardiac angiogenesis, apoptosis, fibrosis, and membrane ionic currents. These miRNAs that are upregulated and/or downregulated could help to differentiate between responders and non-responders to CRT [55]. In patients with AS, miRNAs could be proposed as therapeutic targets or therapeutic agents in the future. However, there are still many steps to be taken, since it is necessary to first determine the cause–effect relationship between miRNA expression and the deterioration of AS.

The GO enrichment analysis of the differentially expressed miRNAs highlighted peptidyl-tyrosine phosphorylation, neuronal cell body membrane, and protein tyrosine kinase activity as key BPs, CCs, and MFs associated with AS. These findings align with a recent report on the cardiovascular side effects of tyrosine kinase inhibitors (TKIs) used to treat chronic myeloid leukemia (CML) [56].

A previous study revealed that nilotinib, a TKI, increased aortic valve thickness and promoted calcification and osteogenic activation in human valvular interstitial cells (VICs) while decreasing autophagy. This effect was linked to the inhibition of the discoidin domain receptor DDR2, which is downregulated in calcified valve tissue [56]. Notably, because protein tyrosine kinase activity is a pathway potentially regulated by miR-143-3p and miR-452-5p, these results suggest that these miRNAs could be regulators of AS.

Biological process analysis revealed that the NRG1/miR-143-3p axis has significant biological importance and potential clinical applications in the context of AS. Previous research has identified miR-143-3p as a regulator of NRG1 [57], a gene critical for cardiac development and function [58]. The association of miR-143-3p with NRG1 was initially explored in Alzheimer’s disease models, where the inhibition of miR-143-3p promoted neuronal survival by targeting NRG1, thus indicating a protective role against cell apoptosis [57]. Translating these findings to cardiovascular research, the involvement of NRG1 in the epidermal growth factor receptor (EGFR) signaling pathway highlights its relevance in cardiac pathologies. Specifically, NRG1 and its receptor ERBB4 have been shown to be associated with LVOT defects, including AS, coarctation of the aorta, and hypoplastic left heart syndrome [59]. These congenital cardiovascular malformations share an embryological origin and a significant genetic component, as demonstrated by the positive association of ERBB4 haplotypes with these defects. Conversely, to date, no studies have demonstrated an interaction between NRG1 and miR-452-5p or between miR-143-3p and miR-452-5p with KLF12 and TMOD2. However, further studies are needed to investigate these potential interactions. Thus, the NRG1/miR-143-3p axis offers a promising avenue for understanding the molecular mechanisms underlying AVS and developing targeted therapies to mitigate the progression of this condition.

Herein, we developed a multiparametric model integrating clinical parameters with miRNAs to enhance diagnostic accuracy. To assess the added value of miRNAs as biomarkers, we first evaluated a clinical-only model based solely on echocardiographic parameters. This model achieved an AUC of 0.887, with an accuracy of 83.58%, a sensitivity of 84.21%, and a specificity of 83.33%. When validated using Weka, the functional logistic classifier yielded an accuracy of 76.3%, a sensitivity of 77%, a specificity of 64.4%, an MCC of 0.456, and an AUC of 0.829. In contrast, the multiparametric model, which integrated clinical variables with miRNAs, demonstrated an improved AUC of 0.954 and an accuracy of 88.89%. The combination of our miRNA set (miR-143-3p and miR-452-5p) with three echocardiographic parameters (LVEF, LV-SV, and GLS) significantly increased the discriminative power of transthoracic echocardiography in clinical practice. Cross-validation further confirmed the robustness of this approach, with the functional logistic classifier achieving a mean accuracy of 78.8%, a sensitivity of 79.3%, a specificity of 70.7%, an MCC of 0.521, and an AUC of 0.837.

These findings suggest that the addition of miR-143-3p and miR-452-5p enhances the diagnostic power of traditional echocardiographic parameters, particularly by improving specificity. This is clinically relevant because AS diagnosis often relies on imaging modalities that may be inconclusive in borderline cases, leading to potential misclassification. The incorporation of miRNAs as complementary biomarkers could help refine patient stratification, particularly in cases where echocardiographic findings alone are ambiguous, thereby improving decision-making for early intervention and risk assessment.

One potential limitation of our study is the relatively small sample size, which raises concerns about possible overfitting in the development of the multi-parametric diagnostic model. To address this, we employed a 10-fold cross-validation strategy during model training and evaluation. Cross-validation is a widely used method to reduce the risk of overfitting by partitioning the dataset into training and validation subsets, ensuring that the model is tested on data not used during its training. This approach allowed us to assess the generalizability of the model and confirm its robustness, as evidenced by the high diagnostic accuracy (78.8%) (AUC: 0.837) observed across the folds. Nonetheless, larger cohorts are needed in future studies to validate these findings further and confirm the applicability of the model in broader populations.

Our diagnostic model includes LVEF, which is a primary determinant of morbidity, mortality, and hospitalization [60]. An accurate assessment of ventricular function is imperative for informed decision-making. Therefore, an LVEF of less than 50% in conjunction with symptomatic AS is an indicator of the need for valve replacement therapy [9]. Subtle changes in decreased LVEF may be an important early indicator of LV dysfunction. Additionally, the LV-SV plays a key role in identifying the type of AS present: (i) high-gradient AS; (ii) low-flow, low-gradient AS with reduced EF; (iii) low-flow, low-gradient AS with preserved EF; and (iv) normal-flow, low-gradient AS with preserved EF [61]. Patients with low flow had worse operative and long-term outcomes than those with normal flow even though they had normal LVEF values [62]. Herrmann et al. [63] reported that an LV-SV < 35 mL/m^2^, but not LVEF, was an independent predictor of 2-year mortality following transcatheter aortic valve replacement. Patients with low-flow, low-gradient AS with preserved EF, and normal-flow, low-gradient AS with preserved EF are particularly challenging to diagnose; consequently, misdiagnoses are common. Therefore, the quantification of GLS has emerged as an imaging marker that describes intramyocardial contractile force and provides information on subclinical myocardial dysfunction due to fibrosis changes [64]. GLS assessment aims to anticipate the early detection of ventricular dysfunction before LVEF deterioration manifests. This technique is considered less dependent on preload and afterload and is more reproducible than LVEF. Moreover, GLS is a sensitive marker of left ventricular (LV) systolic recovery, whereas EF may not be a sensitive marker of LV systolic recovery [65].

The multiparametric score that we propose could help stratify these patients better with a more complete evaluation, especially in discordant cases.

In a study by Coffey et al. [34], echocardiographic measures were related to AS, and lower levels of miR-21-5p and miR-382-5p were found to be significantly correlated with PPG and MPG but not with LEVF or LV-SV. Chen et al. [66] reported a significant correlation between reduced levels of miR-378 and LVH in patients with AS but not with LEVF or LV-SV. With respect to miR-210, only a slight correlation was observed between the left ventricular end-diastolic diameter and the remaining echocardiographic parameters. This research established the prognostic value of higher levels of miR-210 [45]. Fabiani et al. [36] established a correlation between miR-21 and structural and functional deterioration of the left ventricle, as evaluated by echocardiographic GLS. They proposed the evaluation of plasma miR-21 together with GLS for a more accurate estimation of severity in patients with AS with discrepant echocardiographic criteria. Additionally, they speculated on the potential clinical implications of these findings, suggesting that costs could be reduced by limiting the use of expensive magnetic resonance imaging (MRI) [36].

The feasibility of examining miRNAs in clinical practice is challenging and remains to be demonstrated; to date, they are promising targets for future research in elderly patients with AS. Although one of the limitations of this study was the sample size and lack of follow-up, our cohort was consistent with respect to age. It was not possible to perform cardiac MRI on the population to determine the degree of fibrosis.

In conclusion, among older patients with severe AS, we identified two differentially expressed miRNAs, one of which (miR-452-5p) has not been previously described in this population. These findings may open new avenues for research into the molecular pathogenesis of AS. Our multiparametric model that combines biohumeral and functional evaluations could improve the accuracy of AS diagnosis and risk stratification.

Targeting up- or downregulated genes is currently a challenge in molecular biology research focused on seriously interfering with the molecular level of early modification to slow or treat the degeneration of native aortic valves.

Prospective studies in larger populations of patients with severe AS are needed to better understand the prognostic value of this biomarker-functional marker combination model.

## Figures and Tables

**Table 1:** Baseline population characteristics and echocardiographic parameters.

Variables	Control n = 29	AoS n = 58	*p* Value
Demographics (%)			
Age (years) ^a^	82.3 ± 6.4	84.2 ± 8.8	0.451
Sex (male) ^b^	41.4	43.1	0.878
Dyslipidaemia ^b^	34.5	36.2	0.974
Diabetes ^b^	24.1	26.3	0.961
Hypertension ^b^	65.5	81	0.183
Atrial fibrillation ^b^	3.4	32.8	0.002
Smoking habit ^b^	3.4	8.6	0.389
Echocardiography			
LVOTO (mm) ^a^	19.4 ± 5.2	19.7 ± 6.7	0.420
IVS (mm) ^a^	11 ± 3.1	12.9 ± 4.2	0.03
LA diameter (mm) ^a^	34.5 ± 7.8	41.1 ± 15.4	0.03
LVDD (mm) ^a^	42.8 ± 14.4	43.3 ± 18.6	0.654
LVSD (mm) ^a^	24.4 ± 8.3	26.1 ± 11.8	0.083
ePASP (mmHg) ^a^	1.1 ± 1.8	9.5 ± 15.3	0.004
ΔVPeak (m/s) ^a^	1.5 ± 0.5	3.9 ± 1.3	<0.001
PPG	9.5 ± 4.6	67.1 ± 20.3	<0.001
MPG	5 ± 2.6	39.4 ± 11.6	<0.001
AVA (cm^2^) ^a^	2 ± 0.8	0.7 ± 0.3	<0.001
Indexed AVA (cm^2^/m^2^) ^a^	1.2 ± 0.8	0.5 ± 0.3	<0.001
LVOT VTI (cm) ^a^	21.8 ± 7.7	22.3 ± 8.9	<0.001
AV VTI (mm) ^a^	33.1 ± 12.5	92.8 ± 33.4	<0.001
DI (%)	0.7 ± 0.3	0.2 ± 0.1	<0.001
LVEF Teichholz ^a^	73.4 ± 7.5	68.4 ± 9.5	0.02
LVEF Simpson ^a^	72.1 ± 23.9	68 ± 14.6	0.032
Stroke volume (mL) ^a^	37.1 ± 14.1	41.3 ± 16.9	0.09
Global longitudinal strain ^a^	20.7 ± 17.9	17.9 ± 7.9	<0.001
Analytical profile			
GFR (mL/min) ^a^	58.8 ± 18.7	56.9 ± 15.3	0.28
Creatinine (mg/dL) ^a^	0.8 ± 0.3	1 ± 0.5	0,14
NTproBNP (pmol/L) ^a^	94.8 ± 88	1870.7 ± 4653.5	<0.001
Medications (%)			
Diuretics ^b^	13.8	53.4	<0.001
Statins ^b^	24.1	36.2	0.299
Beta-blockers ^b^	13.8	20.7	0.453

Abbreviations: AVA: aortic valve area; AV VTI: aortic valve velocity–time integral; DI: dimensionless index; ePASP: pulmonary artery systolic pressure; GFR: glomerular filtration rate; IVS: interventricular septum; LA: left atrium; LVDD: left ventricular diastolic diameter; LVEF: left ventricular ejection fraction; LVOTO: left ventricular outflow tract; LVOT VTI: left ventricular outflow tract velocity–time integral; LVSD: left ventricular systolic diameter; MPG: mean aortic pressure gradient; NTproBNP: N-terminal pro-brain natriuretic peptide; PPG: peak aortic pressure gradient; ΔVPeak: aortic peak velocity. ^a^: quantitative variables, ^b^: qualitative variables.

**Table 2:** Assessment of the potential diagnostic value of differentially expressed miRNAs and the two-miRNA signature. AUC, area under the curve; CI, confidence interval.

miRNAs	AUC (95% CI)	Sensitivity %	Specificity %	Accuracy %	*p* Value
miR-143-3p	0.690 (0.569–0.812)	53.85	70.15	67.50	0.006
miR-452-5p	0.707 (0.572–0.842)	66.67	77.19	75.76	0.009
miR-143-3p + miR-452-5p	0.763 (0.640–0.886)	66.67	77.36	75.81	0.001

**Table 3:** Sensitivity, specificity, and accuracy of each clinical parameter, and the clinical-only and multiparametric models.

Multiparametric Model	AUC (95% CI)	Sensitivity %	Specificity %	Accuracy %	*p* Value
GLS (%)	0.799 (0.696–0.903)	72.22	76.92	75.71	<0.001
LVEF (%)	0.666 (0.533–0.799)	50.00	69.74	68.29	0.016
Stroke Volume	0.639 (0.512–0.766)	25.00	66.22	64.10	0.047
Clinical-only model	0.887 (0.797–0.978)	84.21	83.33	83.58	<0.001
Multiparametric model	0.954 (0.896–1)	84.62	90.63	88.89	<0.001

AUC, area under the curve; CI, confidence interval; GLS, global longitudinal strain; LVEF, left ventricular ejection fraction.

**Table 4:** GO BP enrichment analysis of the two differentially expressed miRNAs.

Name	Hits	Pop Hits	*p* Value	Adjusted *p* Value
Positive regulation of DNA-templated transcription	16	832	0.0008	0.0038
Cell differentiation	12	599	0.0023	0.0087
Positive regulation of cell population proliferation	11	572	0.0045	0.0142
Negative regulation of DNA-templated transcription	10	649	0.025	0.0475
Peptidyl-serine phosphorylation	8	222	0.0003	0.0025
Intracellular signal transduction	8	413	0.0132	0.0293
Potassium ion transmembrane transport	7	172	0.0004	0.0025
Insulin-like growth factor receptor signaling pathway	6	33	0.0001	0.002
Peptidyl-tyrosine phosphorylation	5	152	0.0061	0.0166
DNA-templated transcription	5	188	0.0139	0.0293

Abbreviations: BP: biological process; DNA: deoxyribonucleic acid; GO: gene ontology.

**Table 5:** GO CC enrichment analysis of the two differentially expressed miRNAs. GO, gene ontology; CC, cellular component.

Name	Hits	Pop Hits	*p* Value	Adjusted *p* Value
Cytoskeleton	9	400	0.0057	0.0142
Glutamatergic synapse	9	449	0.0113	0.0242
Focal adhesion	7	502	0.1054	0.1437
Neuronal cell body membrane	6	32	0.0001	0.001
Voltage-gated potassium channel complex	6	115	0.0004	0.003
RNA polymerase II transcription regulator complex	6	168	0.0027	0.0111
Membrane raft	6	253	0.0169	0.0317
Basolateral plasma membrane	6	267	0.0213	0.0355
Peroxisomal membrane	5	125	0.0037	0.0111
PML body	5	119	0.003	0.0111

**Table 6:** GO MF enrichment analysis of the two differentially expressed miRNAs. GO, gene ontology; MF, molecular function.

Name	Hits	Pop Hits	*p* Value	Adjusted *p* Value
DNA binding	16	988	0.0124	0.0496
DNA-binding transcription factor activity	13	485	0.0004	0.0032
Protein serine/threonine kinase activity	13	646	0.0044	0.0264
DNA-binding transcription activator activity RNA polymerase II-specific	11	533	0.0069	0.0331
Protein tyrosine kinase activity	10	144	0.0001	0.001
Delayed rectifier potassium channel activity	5	48	0.0001	0.0012
Integrin binding	5	184	0.021	0.072
Histone binding	5	200	0.0285	0.0855
Protein kinase activity	5	221	0.0405	0.108
Magnesium ion binding	5	239	0.0529	0.127

## Data Availability

Data transparency is guaranteed. The datasets generated during and/or analyzed during the current study are available from the corresponding author upon reasonable request. We used various software programs for functional enrichment and statistical analysis, all of which are cited in our manuscript.

## Supplementary Materials

The following supporting information can be downloaded at: https://www.mdpi.com/article/10.3390/biomedicines13030671/s1, Table S1: Total filtered RNA-seq read set of plasma circulating miRNAs; Table S2: Top ten most abundant miRNAs detected in the study population.
